# Postinfectious Neurologic Complications in COVID-19: A Complex Case Report

**DOI:** 10.2967/jnumed.120.256099

**Published:** 2021-05-20

**Authors:** Pietro Tiraboschi, Rubjona Xhani, Simone M. Zerbi, Angelo Corso, Isabella Martinelli, Laura Fusi, Giampiero Grampa, Andrea Lombardo, Paola Cavalcante, Cristina Cappelletti, Francesca Andreetta, Alberto Sironi, Alberto Redolfi, Cristina Muscio

**Affiliations:** 1 Neurology 5–Neuropathology Unit, Fondazione IRCCS Istituto Neurologico Carlo Besta, Milan, Italy;; 2 Neurology Department of ASST Lariana, Ospedale Sant’Anna, San Fermo della Battaglia, Italy;; 3 Intensive Care Unit of ASST Lariana, Ospedale Sant’Anna, San Fermo della Battaglia, Italy;; 4 Nuclear Medicine Department of ASST Lariana, Ospedale Sant’Anna, San Fermo della Battaglia, Italy;; 5 Neurology 4–Neuroimmunology and Neuromuscular Disease Unit, Fondazione IRCCS Istituto Neurologico Carlo Besta, Milan, Italy;; 6 Radiology Department of ASST Lariana, Ospedale Sant’Anna, San Fermo della Battaglia, Italy; and; 7 Laboratory of Neuroinformatics, IRCCS Istituto Centro San Giovanni di Dio Fatebenefratelli, Brescia, Italy

**Keywords:** PET, COVID-19, IVIg, SARS-CoV-2, encephalitis, neuroimaging

## Abstract

A 40-y-old woman with severe acute respiratory syndrome coronavirus 2 infection developed neurologic manifestations (confusion, agitation, seizures, dyskinesias, and parkinsonism) a few weeks after the onset of severe acute respiratory syndrome. MRI and cerebrospinal fluid analyses were unremarkable, but ^18^F-FDG PET/CT showed limbic and extralimbic hypermetabolism. A full recovery, alongside ^18^F-FDG normalization in previously hypermetabolic areas, was observed after intravenous immunoglobulin administration.

## PART 1

Severe acute respiratory syndrome coronavirus 2 (SARS-CoV-2) has to date caused about 4,000,000 deaths worldwide. Although typically inducing respiratory symptoms, it is a systemic illness with possible neurologic complications. On the basis of similarities with other coronaviruses, various mechanisms of central nervous system (CNS) damage, ranging from direct CNS infection to dysimmune response (1,2), have been hypothesized for SARS-CoV-2. However, the pathophysiology of neurologic manifestations and the diagnostic role of ancillary investigations remain unclear.

### Case Report

The present case study was authorized by the Ethics Committee of the ASST Lariana, Ospedale Sant’Anna of Como, Italy. Informed written consent was provided for the report publication.

An overweight (body mass index, 28.3 kg/m^2^) 40-y-old woman, otherwise without any risk factors or comorbidities, was transported to the emergency department in late March 2020 (day 0) because of syncope occurring after a few days of fever, anosmia, fatigue, and dyspnea. Arterial blood gas analysis showed severe hypoxemia (PaO_2_/FiO_2_ < 200 mm Hg). Chest CT demonstrated a diffuse interstitial pulmonary pathology with ground-glass opacities. A nasopharyngeal swab was positive for SARS-CoV-2 RNA. After admission to the intensive care unit, the patient was sedated, intubated, and mechanically ventilated.

Early in April (day 14), after sedation discontinuation and extubation, the patient appeared fully alert although episodically agitated and uncooperative. The day afterward, the patient worsened significantly, becoming persistently confused and agitated and showing recurrent generalized tonic–clonic seizures. Brain CT showed unremarkable findings, whereas an electroencephalogram showed bilateral slow waves with epileptiform discharges. Blood chemistry tests revealed a high level of C-reactive protein (62.4 mg/L) and increased white blood cell counts (16.92 × 10^3^/μL). Customary cerebrospinal fluid (CSF) analyses were conversely unremarkable, showing normal cellularity (*n* = 2/μL) and a normal protein content (32 mg/dL). A wide screening for antibodies usually associated with autoimmune encephalitis yielded negative results. CSF antibody testing and reverse-transcriptase polymerase chain reaction testing for most common neurotropic viruses were negative. A SARS-CoV-2 reverse-transcriptase polymerase chain reaction test performed on CSF was also negative, but the CSF was positive for anti-SARS-CoV-2 IgG antibodies and had elevated proinflammatory cytokines.

**FIGURE 1.**
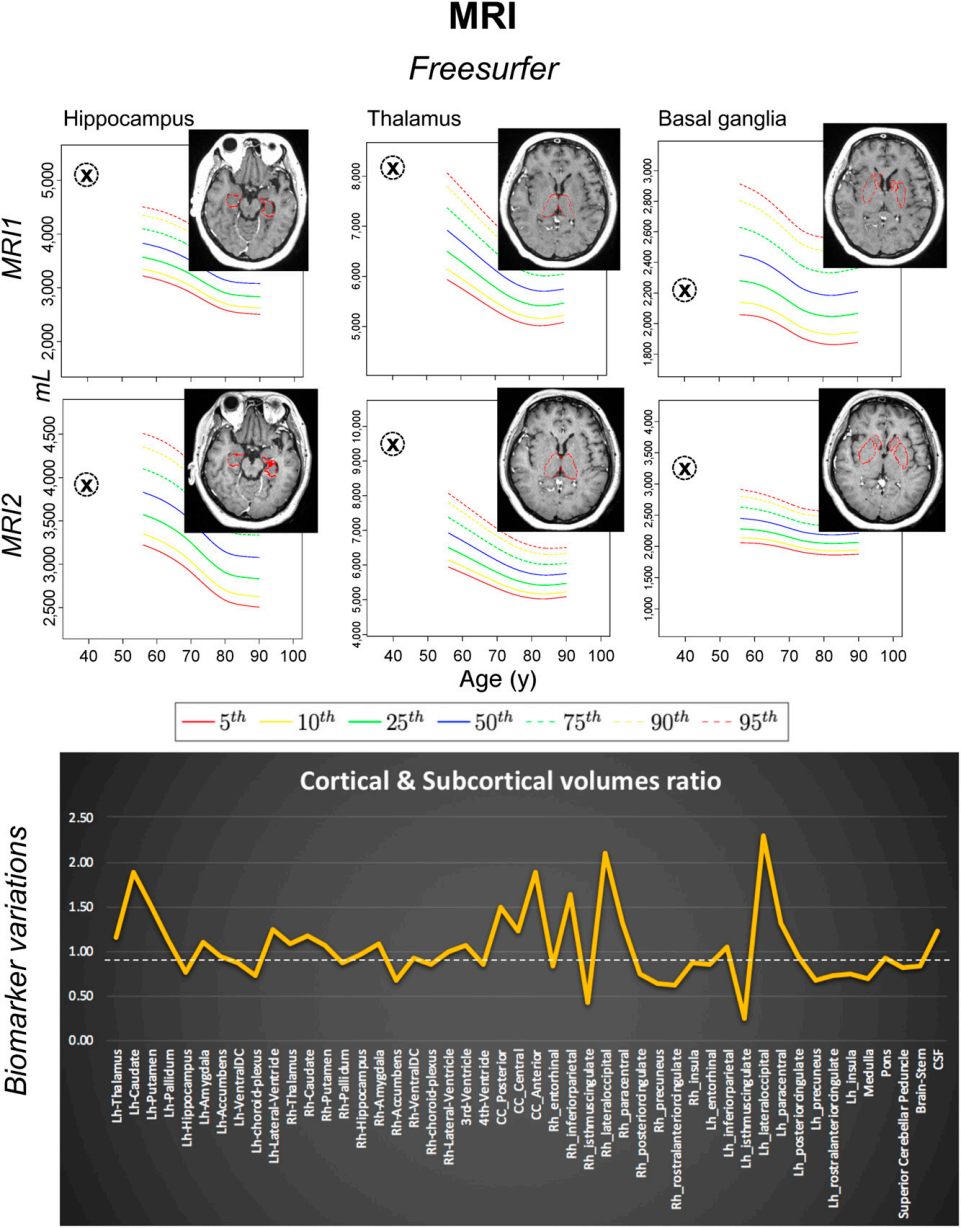
Both MRI1 (day 16) and MRI2 (day 30) were performed before IVIg therapy. T1-weighted 3-dimensional volumetric scans were processed with FreeSurfer software via neuGRID platform (https://www.neugrid2.eu). FreeSurfer percentiles were derived from 532 healthy controls (age range, 55–90 y; mean ± SD, 73.59 ± 6.29 y). No gray matter volume loss was detected. However, biomarker variations graph shows that volumes of most subcortical regions, including amygdala, thalami, and basal ganglia, were on average 10% greater at second time point, possibly reflecting greater edema associated with inflammatory process. Cortical regions showed less homogeneous pattern, but overall, mean cortical volumes at the 2 time points were of similar magnitude. Lh = left hemisphere; Rh = right hemisphere; CC = corpus callosum; DC = diencephalon.

**FIGURE 2.**
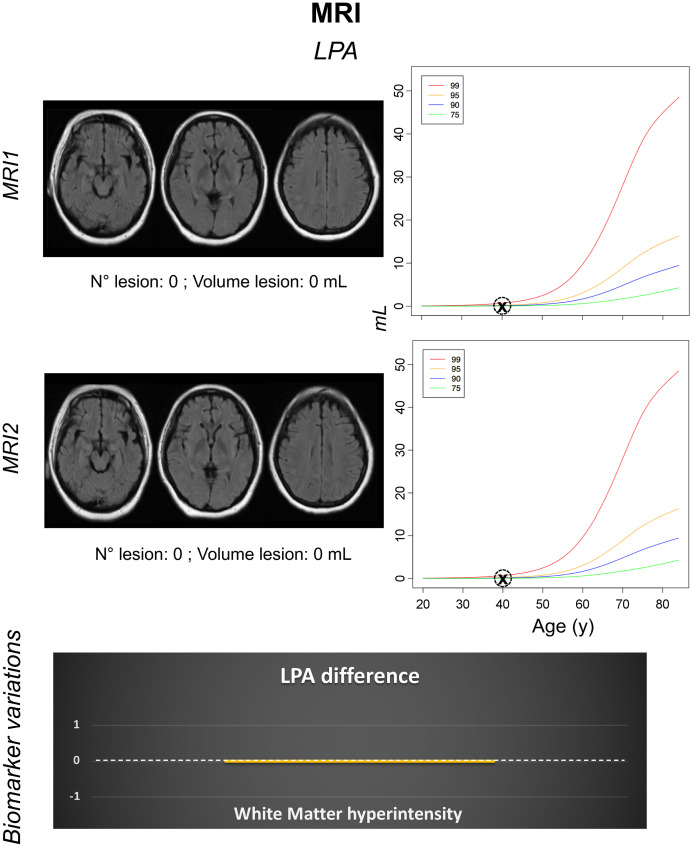
Both MRI1 (day 16) and MRI2 (day 30) were performed before IVIg therapy. Axial 2-dimensional fluid-attenuated inversion recovery MRI scans were processed with lesion prediction algorithm (LPA) via neuGRID platform. LPA percentiles were computed from 629 control subjects (age range, 20–90 y; mean ± SD, 49.82 ± 14.63 y). No white matter hyperintensities were detected.

**FIGURE 3.**
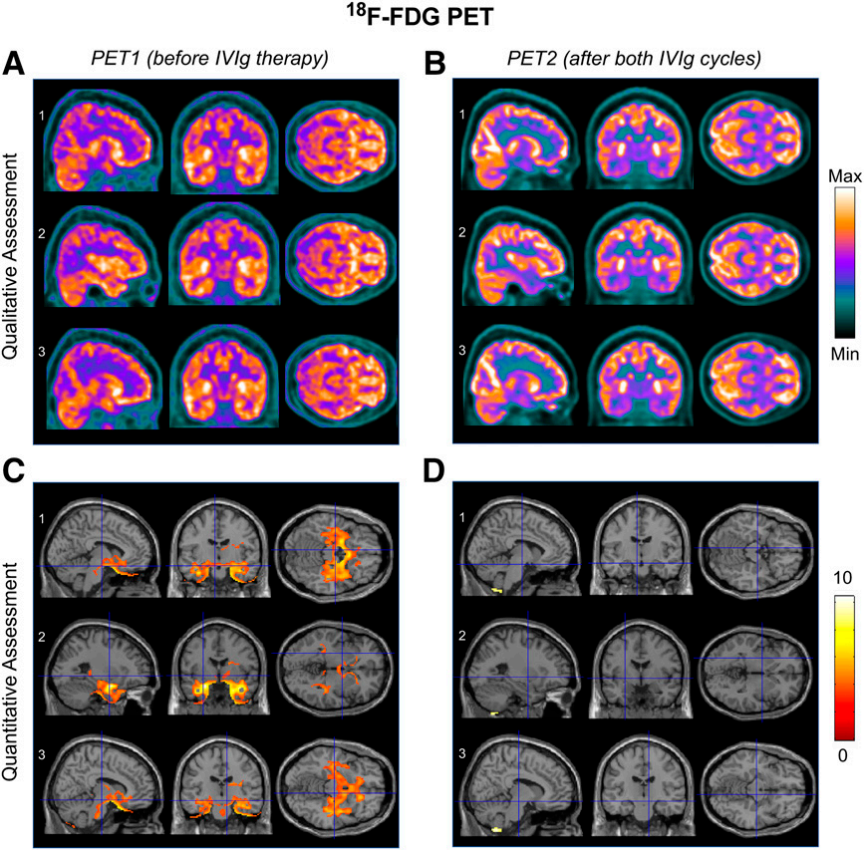
(A and C) PET1 (day 38, closed-eyes condition) showing hypermetabolism in mesial temporal lobes and subthalamic nuclei on both qualitative and quantitative (statistical parametric mapping, SPM12) assessment. (B and D) PET2 (day 143, open-eyes condition) showing areas of increased ^18^F-FDG uptake in parietal-occipital cortex (because of open-eyes condition) on qualitative but not quantitative (SPM) assessment. Mesial temporal lobes and subthalamic nuclei had normal ^18^F-FDG uptake at that time. ^18^F-FDG PET images (A and B) are expressed as total effective counts. Statistical parametric maps are superimposed on T1 template image in Montreal Neurological Institute ICBM 152 brain-template space, describing brain activation by color-coding voxels whose *t* values exceed threshold for significance (*P* < 0.001, extent threshold of 100 voxels, grand-mean-scaling value equal to 6.5 and proportional normalization). SPM12 normative dataset consisted of 53 healthy controls (age range, 20–82 y; mean ± SD, 59.08 ± 10.55 y). Images are shown in neurologic convention. ICBM = International Consortium for Brain Mapping.

Brain MRI (days 16 and 30) had normal findings (Figs. 1 and 2), but cerebral PET/CT (day 38) using a 3-MBq/kg injected activity of ^18^F-FDG revealed increased metabolism in the mesial temporal lobes and subthalamic nuclei (Fig. 3), confirmed by statistical parametric mapping (SPM) quantification (Fig. 3C).

A 5-d intravenous immunoglobulin (IVIg) cycle (0.4 g/kg daily) was started the day afterward. During the first week of treatment, the patient remained confused and uncooperative. She also developed stereotypical paroxysmal lower-limb and choreiform upper-limb movements, for which haloperidol was introduced.

**FIGURE 4.**
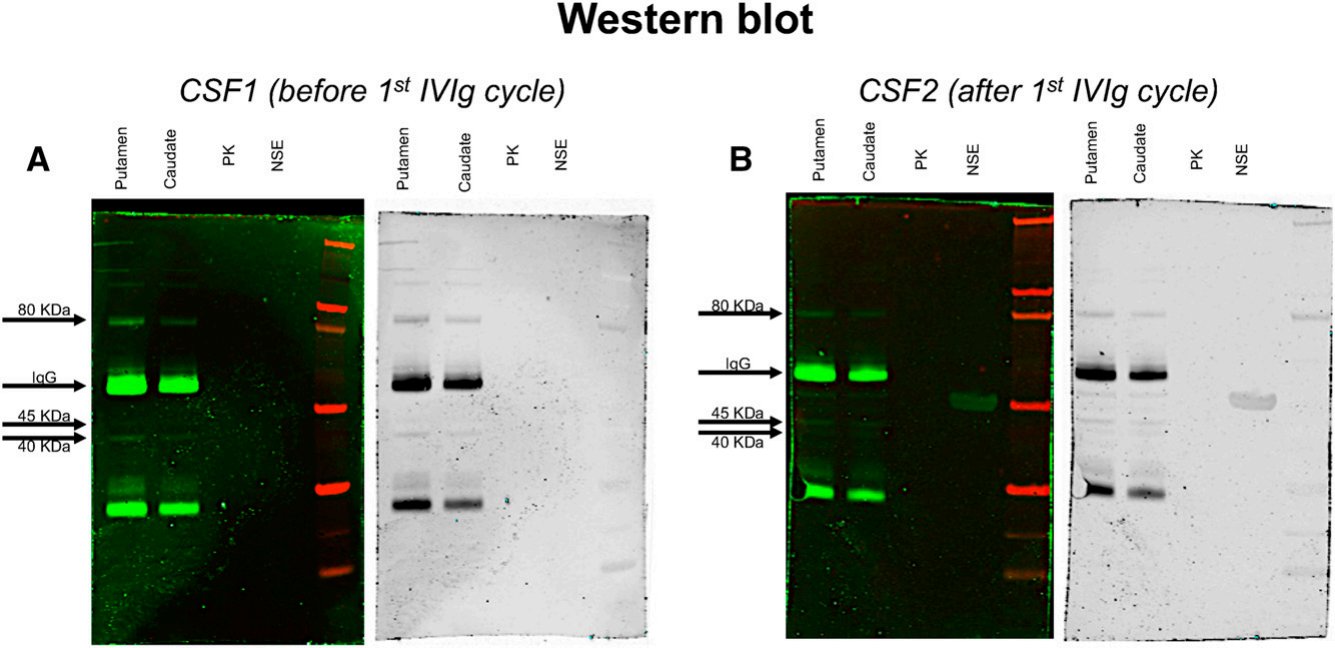
Western blot analyses of total protein extract from human putamen and caudate brain regions (lanes 1 and 2), recombinant human pyruvate-kinase (PK) protein (lane 3), and recombinant human neuron-specific-enolase (NSE) protein (lane 4). Patient’s CSF (A and B) identifies bands with molecular weight of 40, 45, and 80 kDa. A weak positivity for neuron-specific enolase was detected only in post-IVIg sample (B, lane 4).

From the second week onward, the patient rapidly improved. In mid-May (day 57), she was described as fully alert, cooperative, and able to communicate fluently. The abnormal movements earlier described had disappeared. Her neurologic exam revealed exclusively bradykinesia and postural/action tremor of the left upper limb, possibly influenced by the ongoing haloperidol treatment. An electroencephalogram recording at this time showed normal findings. A post-IVIg analysis of CSF showed a reduction of previously increased cytokine levels (interleukine-6, from 47.41 to 0.61 pg/mL; interleukine-8, from 443.33 to 16.71 pg/mL; interleukine-9, from 8.66 to 3.38 pg/mL; and interleukine-15, from 103.13 to 73.22 pg/mL) but, unlike the pre-IVIg CSF (Fig. 4), was immunoreactive to basal ganglia antigens (Fig. 4B). A second IVIg cycle was initiated in late June, when the patient was still displaying parkinsonian signs. Haloperidol was discontinued in late July, after gradual tapering. Two weeks later (day 143), no neurologic signs or significant PET abnormalities were noted any longer (Figs. 3B and 3D).

### Discussion

Although neurotropism has previously been reported for other coronaviruses, evidence for direct CNS infection is lacking for SARS-CoV-2. The present case underlines the possibility of postinfectious inflammatory brain involvement related to SARS-CoV-2, once alternative infectious or autoimmune brain disorders have been excluded.

As also seen recently (3), whereas MRI might be insufficient to reveal cerebral involvement, PET imaging can decisively assist the clinician in making a proper diagnosis and promptly considering appropriate treatment. The observation of ^18^F-FDG hypermetabolism in the subthalamic nuclei may also explain the patient’s dyskinesias, which, to our knowledge, have not yet been reported among possible SARS-CoV-2 complications. Despite conclusions—similar to those in another case study (3)—about the diagnostic role of PET imaging, our report differs from the previous one (3) in several respects. First, we performed imaging and biologic investigations, including an analysis of the cytokine profile, at multiple time points rather than a single time point. Second, our immunologic analyses were based on Western blotting rather than immunostaining. Finally, we used IVIg, instead of steroids, as immunomodulatory treatment. In both studies, however, there was a tight association of clinical with biologic findings (cerebellar syndrome with immunoreactivity against Purkinje cells in the previous study (3); parkinsonism with immunoreactivity against striatal neurons in our study) and a favorable response to immunomodulatory therapy.

Unfortunately, a long-lasting stay of SARS-CoV-2 patients in intensive care units may in itself delay appropriate treatment, since the sedation and intubation required to contrast the effects of the primary process (SARS) can mask the onset of subsequent neurologic complications, making their timely recognition more difficult. A brain disorder was in fact suspected in our patient only after sedation had been discontinued, highlighting the clinical issue of whether—and, if so, when—to consider SARS-CoV-2–related neurologic complications during prolonged periods of sedation and intubation.

The positivity of PET, but not of MRI, in our patient also raises the question of which tools to use for a timely diagnosis, underlining the decisive role of molecular imaging in MRI-negative cases.

## PART 2

### Final Diagnosis

Our final diagnosis was immune-mediated SARS-CoV-2–related encephalitis. Other potential explanations (e.g., paraneoplastic/autoimmune brain disorders) were excluded. However, because the virus was not detected in the CSF, this should be considered a highly probable rather than a confirmed case of SARS-CoV-2–related encephalitis. PET was decisive in revealing direct brain involvement (encephalitis) rather than a mere indirect neurologic consequence of systemic disease (encephalopathy).

Table 1 summarizes the clinical and paraclinical findings that most contributed to clarifying the clinical problem and shedding light on the underlying physiopathology.

**TABLE 1:** Clinical and Paraclinical Findings

Time (d)	Main symptoms/diagnostic tests	Pathogenetic/diagnostic perspectives
0	Systemic and respiratory symptoms; positive swab analysis for SARS-CoV-2 RNA; positive chest CT scan for interstitial pneumonitis	SARS-CoV-2 infection
14–16	Neurologic features (confusion, agitation); normal MRI; normal cellularity and protein content in CSF	Indirect neurologic effects of systemic disease?
15–16	Neurologic features (seizures); slow electroencephalogram with epileptiform discharges; CSF reverse-transcriptase polymerase chain reaction negativity for SARS-CoV-2 but positivity for anti-SARS-CoV-2 antibodies	Direct brain involvement?
15	Negative CSF analysis for neurotropic viruses (herpes simplex-1, herpes simplex-2, human herpes virus-6, varicella-zoster, Epstein-Barr, cytomegalovirus); negative search for antibodies directed against intracellular onconeural (Ma1, Ma2, Hu, Ri, Yo, CV2) or cell surface/synaptic antigens (*N*-methyl-d-aspartate receptor, α-amino-3-hydroxy-5-methyl-4-isoxazolepropionic acid receptor, γ-aminobutyric acid-A receptor, γ-aminobutyric acid-B receptor, contactin-associated proteinlike 2, leucin-rich glioma inactivated 1)	Exclusion of common infectious or paraneoplastic/autoimmune CNS disorders
38	Limbic and extralimbic hypermetabolism on ^18^F-FDG PET	Likely direct brain involvement
82	Neurologic features (parkinsonism); CSF positivity for anti–basal ganglia antibodies	Direct brain involvement of likely immune-mediated etiology
143	Post-IVIg normalization of metabolism on ^18^F-FDG PET	Full recovery after immune-modulatory treatment, further supporting hypothesis of immune-mediated etiology

Absence of the virus in the CSF seems to indirectly support the hypothesis of an immune-mediated etiology. However, in light of the suboptimal sensitivity of the polymerase chain reaction–based method for SARS-CoV-2, failing to identify the virus in the CSF is not sufficient to discard the possibility of a direct viral infection of the CNS. There is also uncertainty about how to interpret positivity for anti-SARS-CoV-2 IgG antibodies in the CSF, potentially indicating either viral CNS penetration with subsequent intrathecal antibody production or passage of peripherally generated antibodies across breakdowns in the blood–brain barrier induced by systemic infection. In addition to the direct CNS infection or, alternatively, the immune activation induced by the virus without CNS invasion, there is a third pathogenetic hypothesis that combines these two, according to which a neurotropic virus that has entered the CNS can activate a postinfectious, immune-mediated encephalitic process. Herpes simplex virus 1 has, for example, been described as a potential trigger for the development of anti-*N*-methyl-d-aspartate receptor autoimmune encephalitis within a few weeks after CNS infection. However, whether this mechanism of action is applicable to SARS-CoV-2 is doubtful, because this virus, unlike herpes simplex virus 1, is not confirmed to have neuroinvasive potential.

Regardless of whether SARS-CoV-2 may have previously invaded the CNS, there are several considerations that, in our patient, argue in favor of a postinfectious, immune-mediated encephalitic process: for example, the 3-wk interval from onset of systemic symptoms to development of neurologic manifestations, the site of PET abnormalities, the detection of anti–basal ganglia antibodies, and the prompt benefit from IVIg therapy.

Although the pathogenesis of most SARS-CoV-2–related complications has not yet been fully elucidated, some authors have proposed an excessive and uncontrolled immune response with massive release of cytokines (the so-called cytokine storm) as a possible additional mechanism of organ damage (4). The CSF cytokine profile of our patient, characterized by a marked concentration of proinflammatory cytokines with subsequent reduction after IVIg treatment, supports a contribution of hyperinflammatory dysregulation to pathogenic events underlying SARS-CoV-2 neurologic complications.

Although movement disorders have rarely been reported in the SARS-CoV-2 context, our patient showed dyskinesias and parkinsonism, both occurring relatively late in the disease course. Because of the concurrent use of haloperidol, it is hard to determine whether the development of parkinsonism was predominantly drug-induced or, conversely, whether haloperidol influenced only its persistence. Likewise, it is difficult to discern what most contributed to its disappearance—whether the impact of the second IVIg cycle or of haloperidol withdrawal. Detection of immunoreactivity against striatal antigens is, however, a strong argument in favor of spontaneous parkinsonism, in line with the results of a recent clinic–radiologic series of neurologic subjects with SARS-CoV-2 infection (5).

Remarkably, the normality of the MRI and conventional CSF analyses indicates that classic investigations might be insufficient to reveal the presence and extent of cerebral involvement in SARS-CoV-2–related encephalitis and that, as in our case, ^18^F-FDG PET could be a more accurate diagnostic option. The present study also highlights the potential utility of ^18^F-FDG PET for follow-up assessment, to better monitor the status of disease and the impact of immunomodulatory treatment.

### Conclusion

This study emphasizes the diagnostic role of ^18^F-FDG PET in an MRI-negative case of SARS-CoV-2–related encephalitis and the still-underreported therapeutic value of IVIg in this context.

## DISCLOSURE

No potential conflicts of interest relevant to this article exist.
